# Deciphering the Mechanisms Underlying the Antitumor Effects of Eucalyptus Essential Oil and Its Component 3-Cyclohexene-1-Methanol Against Human Colon Cancer Cells

**DOI:** 10.3390/ijms26188876

**Published:** 2025-09-12

**Authors:** Sonia Ben Hamouda, Ons Zakraoui, Sonia Souissi, Rania Bouzeyen, Makram Essafi, Khadija Essafi-Benkhadir

**Affiliations:** 1 Institut Pasteur de Tunis, LR16IPT04 Laboratoire d’Epidémiologie Moléculaire et Pathologie Expérimentale, Université de Tunis El Manar, Tunis 1002, Tunisia; soniabenhamoudaa@gmail.com (S.B.H.); ons.zakraoui@ucsf.edu (O.Z.); soniasouissi1997@gmail.com (S.S.); 2 Institut Pasteur de Tunis, LR16IPT02 Laboratoire Transmission, Contrôle et Immunobiologie des Infections, Université de Tunis El Manar, Tunis 1002, Tunisia; rania.bouzeyen@ucsf.edu (R.B.); makramcito@gmail.com (M.E.)

**Keywords:** colon cancer, anti-tumor, cellular effectors, Eucalyptus, essential oil, bioactive compounds

## Abstract

The development of non-toxic, novel anti-tumor alternatives that target key hallmark events of tumor progression is of a high priority for cancer therapy. Natural compounds, such as Essential oils (EOs) derived from plant extracts are a mixture of chemical components known for their diverse pharmacological properties, including anticancer potential. For this purpose, we investigated the antitumor activity of *Eucalyptus globulus* essential oil (EEO) and its major constituents against colorectal cancer cells in vitro. EEO significantly reduced the viability of colon cancer LS174 cells, induced caspase-dependent apoptosis and triggered cell cycle arrest by modulating the expression of several effectors involved in these processes. Mechanistically, EEO exhibited its activity by targeting p38, SAPK/JNK, ERK1/2, and AKT kinases in LS174 cells. Considering the pivotal role of p53 status in mediating the response to anticancer therapies, we further investigated the effects of Eucalyptol, 3-Cyclohexene-1-methanol, α-Pinene, and α-Terpineol, identified as major components of EEO, on the viability of human colon adenocarcinoma LS174 (wild type p53) and HT29 (mutant p53) cell lines. Interestingly, we highlighted for the first time that 3-Cyclohexene-1-methanol exhibited the most anti-proliferative activity against both tumor cells irrespective to their p53 status. It exerted its effect by inducing apoptotic cell death, disturbing cell cycle progression along with reducing the phosphorylation of key components of the proliferation and survival pathways p38, ERK1/2, and AKT kinases. Our results suggest that Eucalyptus essential oil and its component, 3-Cyclohexene-1-methanol represent promising multi-targeting candidates for colorectal cancer therapy.

## 1. Introduction

Colorectal cancer (CRC) remains a major public health issue worldwide due to its high prevalence and impact on morbidity and mortality. According to GLOBOCAN data, in 2022, CRC ranked third in incidence and second in mortality with more than 1.92 million new cases and over 900,000 deaths [1]. In some situations, despite improved progression-free survival mediated by advancements in treatment strategies, relapses remain common, mainly due to late-stage diagnosis and refractoriness or acquired resistance of CRC patients to available therapies. These limitations are also challenged by the adverse effects of conventional drugs [2,3]. Therefore, identifying alternative therapies that are less toxic to healthy cells [4] and developing more effective cancer therapy options to overcome the current treatments limitations are crucial. The discovery of natural plant-derived compounds is a significant achievement in the history of health care. Over the years, their use alone or in combination with synthetic drugs has effectively resolved many health concerns [5,6]. Accordingly, we have previously shown that Kaempferol, a phenolic compound from quince plant, either alone or in combination with the conventional 5-Fluorouracil (5-FU), could reverse the chemoresistance in colon cancer cells, increasing their sensitivity to chemotherapy [7]. Over time, significant progress has been made in the extraction of plant secondary metabolites, such as essential oils (EOs) known by their aroma and volatility [8]. EOs are complex mixture of various chemical compounds, mainly consisting of terpenes and phenylpropanoids, which are responsible for a wide range of biological and pharmaceutical properties [9]. The literature has discussed the diverse benefits of EOs, including antimicrobial, anticancer, antioxidant, antiparasitic, insecticidal, anti-inflammatory, viricidal, fungicidal, wound healing, antihypertensive, and analgesic properties [8]. Therefore, using EOs in cancer therapies has garnered considerable attention globally. Several studies have shown that essential oils from different plant species possess interesting anti-tumor properties [6]. They could be used as a complement or alternative to conventional cancer treatments. Indeed, EOs can exert anti-proliferative effects through different mechanisms, including disruption of the cell membrane and induction of apoptosis [6,9]. However, as potential leads for anti-tumor drug discovery, their translation from bench to clinic is facing several challenges relative to their pharmacokinetics and pharmacodynamics. Recently, nanoformulation approaches of essential oils have emerged as a promising strategy to enhance their bioavailability, stability, and therapeutic efficacy in various diseases notably cancer. Indeed, the use of drug delivery system can bypass the limitations posed by the hydrophobicity of these extracts making them valuable in pharmaceutical, cosmetic, and agrochemical applications [10,11].

The genus Eucalyptus (E.) is well known worldwide for its medicinal properties [12]. This genus is native to Australia and Tasmania and belongs to the Myrtaceae family with over 900 different species and subspecies. Eucalyptus trees are cultivated in various regions around the world with subtropical and Mediterranean climates [13,14]. Certain species, such as *E. polybractea*, *E. smithii*, and *E. globulus*, are known for their essential oils, which are widely used in pharmaceutical and cosmetic products. The EOs obtained from Eucalyptus exhibit multiple benefits, including anti-inflammatory, anti-immunomodulatory, antioxidant, anticancer, and wound-healing properties [15]. In Tunisian folk medicine, Eucalyptus sp. essential oil has traditionally been used to treat respiratory tract disorders such as pharyngitis, bronchitis, and sinusitis [16]. Several studies have shown that essential oils extracted from Eucalyptus Sp. exert anti-tumor effects in various cancer cell types including colorectal cancer [17,18,19,20], suggesting that bioactive components that can serve as effective agents for cancer treatment, either alone or in combination with conventional drugs. In this study, we investigated the anti-proliferative properties of *Eucalyptus globulus* essential oil sourced from Tunisia and its major bioactive constituents to assess their potential use as anti-tumor agents.

## 2. Results

### 2.1. EEO Inhibits the Viability of LS174 Colon Cancer Cells

To assess the effect of EEO on the viability of colon cancer cells, we treated the human colon adenocarcinoma LS174 cells with increasing concentrations of the essential oil (0.012, 0.025, 0.05, 0.1, 0.2, 0.4, and 0.8 µg/mL) for 24 h. The MTT assay revealed that EEO significantly reduced the viability of LS174 cells in a dose-dependent manner, compared to mock-treated cells. At a concentration of 0.1 µg/mL, the percentage of inhibition was estimated to 33%, which further increased to 55% at 0.2 µg/mL reaching approximately 100% of inhibition at concentrations exceeding 0.2 µg/mL (0.4 and 0.8 µg/mL).

**Figure 1:**
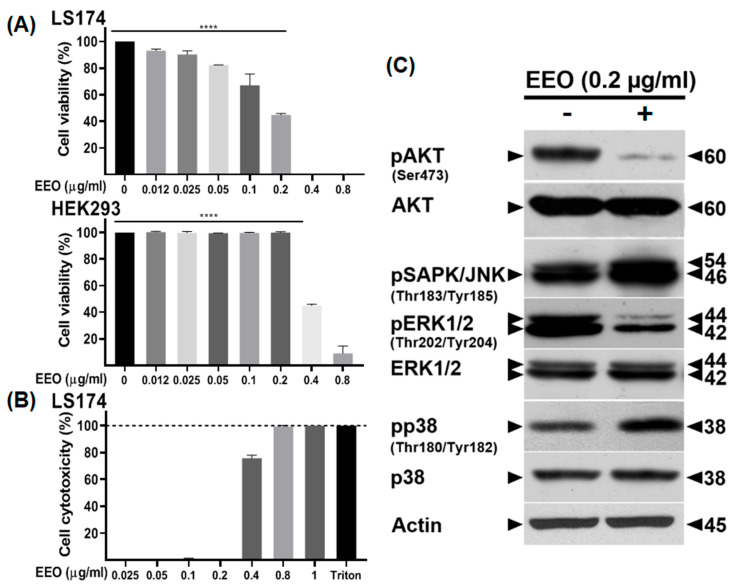
Eucalyptus essential oil (EEO) affects the viability of human colon adenocarcinoma LS174 cells by targeting multiple survival signaling pathways. LS174 and Human Embryonic Kidney 293 (HEK293) cells were cultured in 96-well plates and treated with vehicle (DMSO) or increasing concentrations of EEO (0.012, 0.025, 0.05, 0.1, 0.2, 0.4, and 0.8 µg/mL) for 24 h. (**A**) Cell viability was assessed using the MTT assay, with absorbance measured at 540 nm. (**B**) Cytotoxicity was evaluated by lactate dehydrogenase (LDH) release assay. Culture supernatants from LS174 cells treated with EEO or vehicle (DMSO) were analyzed, and results were expressed as a percentage of LDH release induced by 1% Triton X-100 (100% Cytotoxicity). (**C**) Whole-cell lysates were subjected to SDS-PAGE and immunoblotted with the indicated antibodies. Actin was used as a loading control. Data represent the mean ± standard error (SE) of three independent experiments. Statistical significance was determined using one-way ANOVA (**** *p* < 0.0001 vs. control).

To determine whether EEO is selective toward colon cancer cells without exhibiting a cytotoxic effect against normal cells, we further investigated its effect on the non-tumorigenic Human Embryonic Kidney 293 (HEK293) cells as the control. The MTT assay showed that the viability of HEK293 cells remained unaffected when treated with EEO at the concentration of 0.2 µg/mL with inhibitory effect observed at a concentration of 0.4 µg/mL and higher (Figure 1A).

To better characterize the effect of EEO on LS174 cells and check whether it did not affect cell membrane integrity reflected by the release of cytosolic lactate dehydrogenase (LDH), treated and mock cells were subjected to LDH assay after 24 h.

The results showed that EEO concentrations between 0.025 and 0.2 µg/mL did not induce any detectable toxicity in LS174 cells when compared to the positive control, Triton, which caused 100% toxicity. Notably, a toxic effect was observed at a concentration of 0.4 µg/mL and reached 100% at 0.8–1 µg/mL of EEO (Figure 1B).

Based on these results, we selected a dose of 0.2 µg/mL of EEO corresponding to the half-maximal inhibitory concentration (IC_50_) value and exhibiting no cytotoxic effects toward non-tumorigenic cells for subsequent mechanistic studies.

### 2.2. EEO Modulates Survival-Signaling Pathways in LS174 Colon Cancer Cells

To further characterize the mechanisms underlying the inhibitory effect of EEO on the proliferation of LS174 cells, we analyzed the activation of kinases known to play critical roles in the development and progression of colorectal cancer [21]. Western blot analysis showed decreased phosphorylation of Extracellular-Regulated Kinase 1/2 (ERK1/2), p38 (Mitogen-activated protein kinase) MAPK, and AKT (Protein kinase B) along with increased phosphorylation of Stress-activated protein kinase/c-Jun NH(2)-terminal kinase (SAPK/JNK) in EEO-treated LS174 cells compared to mock-treated cells (Figure 1C).

Our results suggest that EEO-mediated anti-proliferative activity against LS174 colon cancer cells involves the modulation of cell survival signaling pathways.

### 2.3. EEO Induces Cell Cycle Arrest in Colon Cancer LS174 Cells

**Figure 2:**
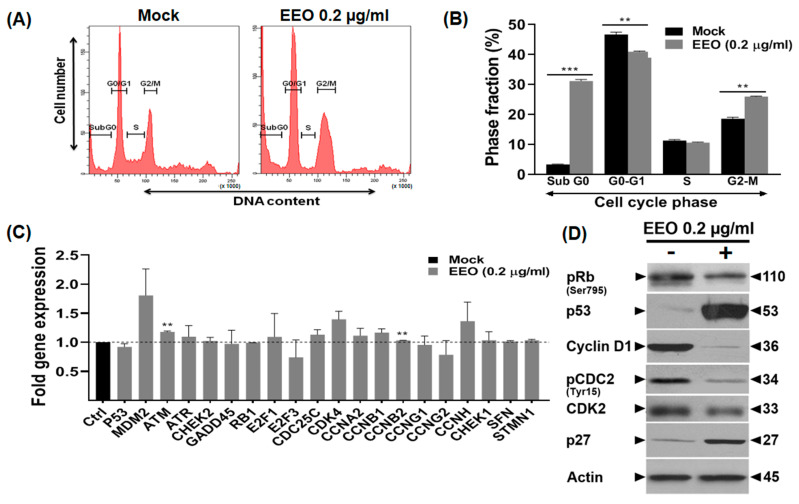
EEO induces cell cycle arrest in human colon adenocarcinoma LS174 cells by targeting various cell cycle regulatory proteins. Exponentially growing LS174 cells were treated with EEO (0.2 µg/mL) or vehicle (DMSO) for 24 h. (**A**) Cell cycle distribution was analyzed by flow cytometry following propidium iodide staining. (**B**) Quantification of cell populations in G_0_/G_1_, S, and G_2_/M phases is presented as percentage values. (**C**) The levels of mRNA transcripts were quantified by real time PCR. The control values were normalized to 1. (**D**) Whole cell lysates were analyzed on SDS-PAGE gel and probed with the indicated antibodies for Western blot analysis. Actin was used as loading control. Data represent the means ± SE of three separate experiments. Statistical differences were analyzed with Student’s *t*-test (** *p* < 0.01, and *** *p* < 0.001 as compared to the control).

We asked whether the decrease in cell numbers was associated with cell cycle disruption. As shown in Figure 2A,B, analysis of the cell cycle progression phases after 24 h of EEO (0.2 µg/mL) treatment indicated that, compared to mock-treated cells, the number of LS174 cells in the subG0 phase significantly increased from 3.3% (±0.14%) to 31.15% (±0.49%). However, the percentage of LS174 cells decreased in the G0-G1 phase (from 46.65% (±0.78%) to 40.95% (±0.21%)) and S phase (from 11.25% (±0.35%) to 10.6% (±0.14%)), along with an increase in the percentage of G2-M population (from 18.55% (±0.49%) to 25.95% (±0.21%)). This result suggests that EEO decreased LS174 cell viability by inducing cell cycle arrest in G2-M phase.

To further unravel the mechanisms underlying EEO-induced cell cycle arrest, we analyzed the expression of key effectors regulating this process by qPCR and immunoblotting. As shown in Figure 2C, EEO slightly increased the expression of Ataxia-Telangiectasia Mutated (ATM), Mouse Double Minute 2 homolog (MDM2), Ataxia Telangiectasia and Rad3-related protein (ATR), E2F transcription factor 1 (E2F1), Cell Division Cycle 25C (CDC25C), Cyclin Dependent Kinase 4 (CDK4), Cyclin A2 (CCNA2), Cyclin B1 (CCNB1), Cyclin B2 (CCNB2), and Cyclin H (CCNH) transcripts. In addition, it modestly affected the mRNA levels of E2F transcription factor 3 (E2F3), Cyclin G2 (CCNG2), and p53. Hence, we investigated the protein levels of p53, the cyclin-dependent kinase inhibitor p27 (p27), cyclin D1, Cyclin Dependent Kinase 2 (CDK2), and the phosphorylated forms of Cell Division Cycle 2 (pCDC2) and Retinoblastoma protein (pRb). We found that EEO treatment increased the expression of p53, which correlated, with an up-regulation of p27. Conversely, the expression levels of cyclin D1, CDK2, and phospho Rb and CDC2 were decreased after 24 h of EEO treatment. Overall, these findings suggest that EEO induces G2-M phase arrest in LS174 colon cancer cells by modulating key cell cycle-regulatory proteins and cyclin-dependent kinase inhibitors.

### 2.4. EEO Induces Caspase-Dependent Apoptosis in LS174 Colon Cancer Cells

**Figure 3:**
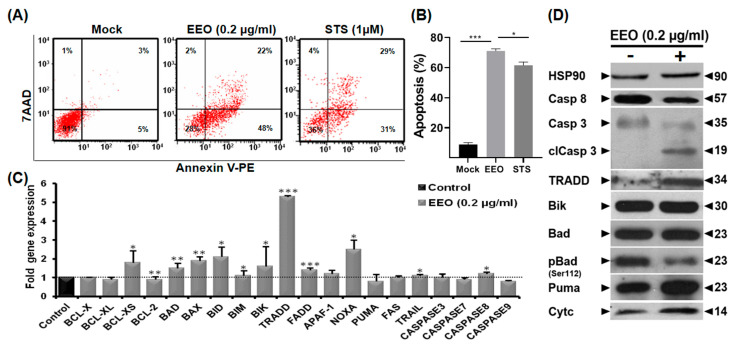
EEO induces a caspase-dependent apoptosis in LS174 colon cancer cells. LS174 cells were treated with vehicle (DMSO) or EEO (0.2 µg/mL) for 24 h. (**A**) Flow cytometric analysis of apoptotic cell death using Annexin-V/7-AAD staining. Staurosporine (1 µM) was used as a positive control for apoptosis. Lower left quadrant (Annexin V^−^/7-AAD^−^) represents live cells. Lower right quadrant (Annexin V^+^/7-AAD^−^) represents early apoptotic cells, upper right quadrant (Annexin V^+^/7-AAD^+^) represents late apoptotic cells and upper left quadrant (Annexin V^−^/7-AAD^+^) represents cells death by necrosis. (**B**) Histogram summarizing the percentages of both early and late apoptotic cells. (**C**) mRNA transcript levels were quantified by real-time PCR, with control values normalized to 1. (**D**) Whole-cell lysates were resolved by SDS-PAGE and analyzed by Western blotting with the indicated antibodies. Actin served as a loading control. Data are presented as the mean ± SE from three separate experiments. Statistical differences were analyzed using Student’s *t*-test (* *p* < 0.05, ** *p* < 0.01, and *** *p* < 0.001 as compared to the control).

The fact that EEO treatment led to the inhibition of LS174 cell viability along with morphological changes and an increase in the sub-G0 population suggest that these cells might have undergone apoptosis. To characterize this process, we quantified the percentage of apoptotic cells by flow cytometry following Annexin-V staining. As shown in Figure 3A,B, EEO at a concentration of 0.2 µg/mL induced a significant increase in the percentage of apoptotic LS174 cells (71%) compared to mock-treated cells. This result suggests that the inhibition of LS174 cell viability by EEO is concomitant to the induction of apoptotic cell death. Hence, to better elucidate the mechanisms underlying this process, we investigated the impact of EEO on several cellular effectors by measuring the expression levels of some genes related to apoptotic cell death. EEO treatment led to up-regulation in the expression of pro-apoptotic B-cell lymphoma 2 (BCL-2) interacting mediator of cell death (BIM), BCL2-interacting killer (BIK), BCL-2-associated agonist of cell death (BAD) and Bcl-2–associated X protein (BAX) genes, in LS174-treated cells. We also noticed an increase in the expression of the TNFR1-associated death domain protein (TRADD) and Fas-associated death domain protein (FADD) along with a significant up-regulation of TNF-related apoptosis-inducing ligand (TRAIL). In addition, EEO increased the expression of caspase 8, and two inhibitors of the anti-apoptotic protein BCL-2, the Phorbol-12-myristate-13-acetate-induced protein 1 (NOXA) and BCL-XS transcripts (Figure 3C). These findings suggest that EEO activates the mitochondria-mediated apoptotic pathway by changing the balance between pro-apoptotic and anti-apoptotic genes.

To validate the expression of regulated genes at the protein level, we performed immunoblot analysis focusing on key apoptotic regulators. EEO induced the cleavage of caspase-3, decreased the pro-caspase 8 and reduced the phosphorylated form of BAD while up-regulating levels of p53 upregulated modulator of apoptosis (Puma), cytochrome c and TRADD proteins. No effect was observed on the pro-apoptotic Bik protein (Figure 3D). As expected, we did not detect Bax protein in whole cell lysates, consistent with previous reports that LS174 cells harbor mutations in the Bax gene, which is a non-sense transcript, resulting in its rapid degradation due to non-sense mediated decay [22].

Altogether, our results suggest that EEO triggers apoptosis in LS174 cells in a caspase-dependent mechanism.

This effect may result from the activity of minor or major EEO components or from their combined action.

### 2.5. Characterization of the Chemical Composition of Eucalyptus Essential Oil

The chemical characterization of the EEO, carried out by gas chromatography coupled to mass spectrometry (GC/MS), identified twenty constituents representing 94.4% of the total EEO extract (Table 1). Their retention indices (n-alkanes (C9–C24)) were used as reference points in calculating the relative retention indices, and their percentage composition is listed in Table 1. Six hydrocarbon constituents (including three sesquiterpenes) and six oxygenated compounds (only one being a sesquiterpene) were detected.

The chemical analysis of EEO composition indicated that it comprises four main chemical groups accounting for 80.14% (Table 1). The first class contained oxygenated monoterpenes (68.6%), and the main compounds were eucalyptol (63.28%), Z-citral (0.12%), α-Terpineol (4.95%), 2,6-octadienal (0.12%), and Geraniol (0.13%). The second class of this essential oil corresponded to hydrocarbon monoterpenes (10.5%), which were dominated mainly by α-pinene (9.19%), β-pinene (0.25%), and α-phellandrene (1.06%). The hydrocarbon sesquiterpenes such as caryophyllene (0.44%), α-caryophyllene (0.04%), and Bicyclogermacrene (0.41%) constituted the third class. Finally, we found the fourth class belonging to the oxygenated sesquiterpenes containing 0.15% Ledol. The remaining components (14.35%) included Butanal (1.29%), 1,2,6-Octariene (0.65%), 1,4-Cyclohexadiene (0.32%), benzene (1.08%), cyclohexene (0.45%), 1-H-Cyclohexene-1-one (0.21%), 3-Cyclohexene-1-methanol (10.18%), and Cis-p-Mentha-1(7) (0.17%).

Thus, the major EEO compounds were eucalyptol known as 1,8-cineole (63.28%), 3-Cyclohexene-1-methanol (10.18%), α-pinene (9.19%), and α-terpineol (4.95%).

### 2.6. The Major Bioactive Components of EEO Inhibit the Proliferation of LS174 and HT29 Colon Cancer Cell Lines

The 3-cyclohexene 1-methanol has been identified in the composition of EEO as one of the four major constituents. To our knowledge, this compound has never been investigated for its anti-tumor effects. Therefore, we asked whether it could affect the viability of colon cancer cells in vitro, compared to α-Pinene, Eucalyptol and α-Terpineol bioactive molecules that have already been reported to exhibit anticancer activities against numerous cancer cell lines [11,23,24].

The tumor suppressor p53 plays a critical role in regulating tumor progression [25]. In CRC, mutations in the TP53 gene were observed in approximately 60% of patients [26]. Given that the total EEO extract reduced the viability of LS174 cells, which express wild-type p53, we sought to investigate whether its major compounds could impact the proliferation of cells harboring p53 mutations, which are usually associated with the transition from adenoma to invasive carcinoma in CRC [27]. To this end, we assessed the effect of the four identified major compounds on the viability of both human colon adenocarcinoma LS174 (wildtype p53) and HT29 (p53 mutant) cell lines. After 24 and 72 h, the treatment of LS174 and HT29 cells with concentrations ranging from 0.01 µg/mL to 2.8 µg/mL of the selected compounds induced varying effects depending on the molecule and the used concentration.

**Figure 4:**
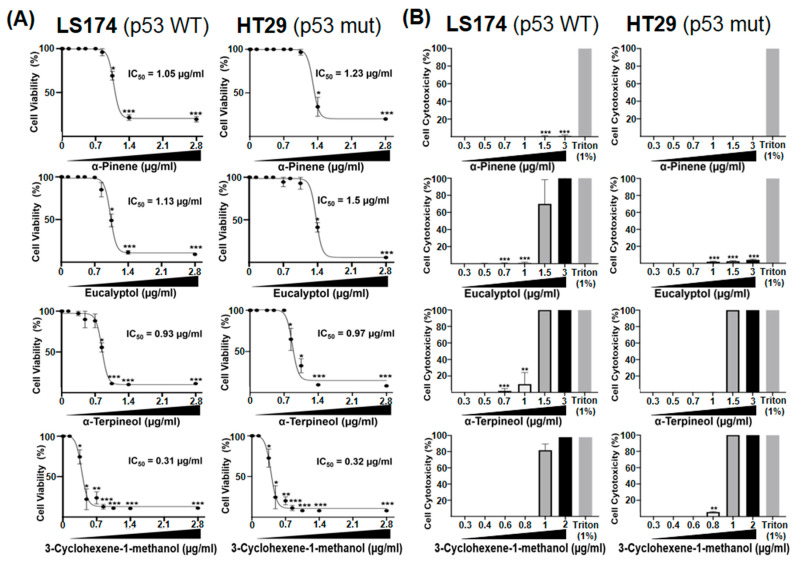
The four major compounds of Eucalyptus essential oil (EEO) inhibit the proliferation of LS174 and HT29 colon cancer cells independently of their p53 status. LS174 and HT29 cells were treated for 72 h with increasing concentrations of α-pinene, eucalyptol, α-terpineol, and 3-Cyclohexene-1-methanol or vehicle (DMSO). (**A**) Cell viability was assessed using the MTT assay. (**B**) Cytotoxicity was evaluated by lactate dehydrogenase (LDH) release assay. LDH activity was expressed as a percentage of the release induced by 1% Triton X-100 (100% cyototoxicity). Data represent the mean ± standard error (SE) of three independent experiments. Statistical significance was determined using Student’s *t*-test (* *p* < 0.05, ** *p* < 0.01, *** *p* < 0.0001 vs. control).

We found that α-Pinene did not significantly affect the viability of HT29 and LS174 cells after 24 h of treatment, with IC50 values exceeding 2.7 µg/mL (Table 2). In contrast, Eucalyptol, α-Terpineol, and 3-Cyclohexene-1-methanol moderately reduced HT29 cell viability with IC50 values of 1.27 µg/mL, 1.19 µg/mL, and 0.76 µg/mL, respectively. While the IC50 of Eucalyptol exceeded 3.09 µg/mL, α-Terpineol and 3-Cyclohexene-1-methanol induced comparable inhibitory effects in LS174 cells with IC50 values of 0.85 µg/mL and 0.6 µg/mL, respectively. After 72 h of treatment, all tested compounds exhibited enhanced antiproliferative effects on both cell lines, independent of p53 status (Figure 4A). In HT29 cells, IC50 values were 1.23 µg/mL, 1.5 µg/mL, 0.97 µg/mL, and 0.32 µg/mL for α-Pinene, Eucalyptol, α-Terpineol, and 3-Cyclohexene-1-methanol, respectively. Similarly, in LS174 cells, the IC50 values were 1.05 µg/mL, 1.13 µg/mL, 0.93 µg/mL, and 0.31 µg/mL for the same constituents, respectively (Figure 4A, Table 2). At these concentrations, inhibition rates exceeded 50% in all cases, reaching over 80–90% at the highest concentrations. These results highlight that 3-Cyclohexene-1-methanol exhibited the best antiproliferative activity on both HT29 and LS174 cells, with an IC50 of 0.3 µg/mL.

To verify whether the inhibitory effect on HT29 and LS174 cell viability induced by the tested compounds was associated with cytoxicity, we performed the LDH release assay to quantify the lactate dehydrogenase release as a consequence of the loss of membrane integrity. Compared to the positive control of 100% toxicity induced by Triton, we found that 24 h treatment of HT29 cells with α-Pinene and Eucalyptol did not induce LDH release (Figure S1). Similarly, treatment of cells with α-Terpineol and 3-Cyclohexene-1-methanol at concentrations below 1 µg/mL was not associated with cell toxicity. However, concentrations above 1 µg/mL induced 100% toxicity (Figure 4B).

Treatment of LS174 cells with increasing concentrations of α-Pinene for 24 h did not induce cell toxicity. Similarly, Eucalyptol, α-Terpineol, and 3-Cyclohexene-1-methanol were not toxic at concentrations below 1 µg/mL. However, cell treatments with 1 µg/mL and 3 µg/mL induced cytotoxic effects (Figure S1).

After 72 h, treatment with a range of concentrations below 3 µg/mL, α-Pinene and Eucalyptol were not toxic to HT29 cells compared to α-Terpineol and 3-Cyclohexene-1-methanol. However, concentrations exceeding 1.5 µg/mL for α-Terpineol and 1 µg/mL for 3-Cyclohexene-1-methanol resulted in 100% cytotoxicity. In LS174 cells, α-Pinene did not cause cytotoxicity after 72 h, whereas Eucalyptol (1.5 µg/) and 3-Cyclohexene-1-methanol (1 µg/mL) induced 70% and 83% toxicity, respectively, that reached 100% at a concentration above 1.5 µg/mL (Figure 4B).

The four components of EEO, α-Pinene, Eucalyptol, α-Terpineol, and 3-Cyclohexene-1-methanol, exhibited an inhibitory effect on the viability of both tumor cell lines, particularly after 72 h of treatment. However, at higher concentrations, this activity was associated with significant cytotoxicity. Our results demonstrate for the first time that 3-Cyclohexene-1-methanol is endowed with strong antiproliferative activity against colorectal tumor cells regardless of p53 status, with a safe IC50 of approximately 0.3 µg/mL.

### 2.7. 3-Cyclohexene-1-Methanol Compound Induces Cell Cycle Arrest in Colon Cancer Cells

**Figure 5:**
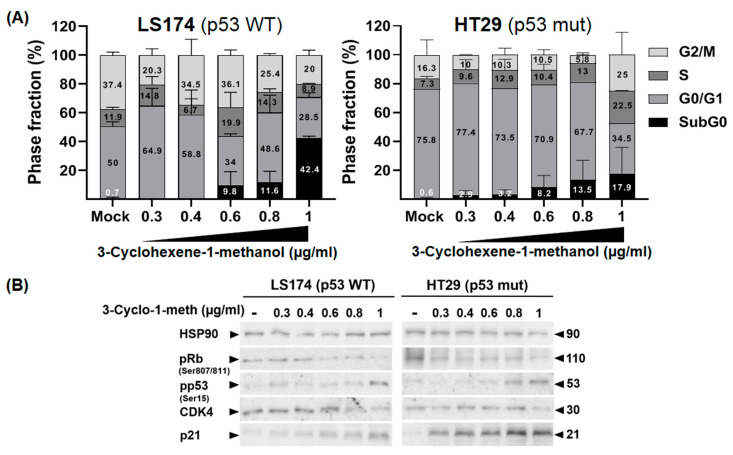
3-Cyclohexene-1-methanol induces cell cycle arrest in colon cancer cells. Exponentially growing LS174 and HT29 cells were treated with increasing concentrations of 3-Cyclohexene-1-methanol (0.3, 0.4, 0.6, 0.8, and 1 µg/mL) or with vehicle (DMSO) for 72 h. (**A**) Cell cycle distribution was analyzed by flow cytometry following propidium iodide (PI) staining. The histogram illustrates the relative proportions of cells in the sub-G_0_, G_0_/G_1_, S, and G_2_/M phases of the cell cycle. (**B**) Whole-cell lysates were resolved by SDS-PAGE and analyzed by Western blotting using the specified antibodies. HSP90 was employed as a loading control.

To further characterize the cellular mechanisms by which 3-Cyclohexene-1-methanol compound inhibits the proliferation of HT29 and LS174 cells, we first evaluated its impact on cell cycle progression using a range of concentrations (0.3 µg/mL to 1 µg/mL). As shown in Figure 5A, we found that the 3-Cyclohexene-1-methanol treatment induced notable perturbations in the cell cycle of both colorectal cancer cell lines.

In HT29 cells, the compound led to an accumulation of cells in the G0/G1 phase (from 75.8% to 77.4% at 0.3 µg/mL) and in the S phase (from 7.3% to 9.6% and 12.9% at 0.3 and 0.4 µg/mL, respectively) suggesting a G1-S phase arrest. This effect was accompanied by a decrease in the G2/M phase population (from 16.3% to 10% and 10.3%, respectively). However, at 0.6 µg/mL and 0.8 µg/mL, a distinct shift was observed, with a reduction in G0/G1 (from 75.8% to 70.9% and 67.7%, respectively) and G2/M (from 16.3% to 10.5% and 5.8%, respectively) populations. Concomitantly, these doses induced cell cycle arrest in the S phase where the percentage of cells increased from 7.3% to 10.4% and 13%, respectively. In LS174 cells, the compound induced cells arrest in G0/G1 phase (from 50% to 64.9% and 58.8%, respectively) at concentrations of 0.3 µg/mL and 0.4 µg/mL. In contrast, higher doses (0.6 µg/mL, 0.8 µg/mL) led to an accumulation of cells in the S phase (from 11.9% to 19.9%, and 14.3%, respectively) and in sub-G0 phases (from 0.7% to 9.8%, and 11.6%, respectively, reaching 42.4% for the concentration of 1 µg/mL), indicative of induced cell apoptosis. Additionally, increasing concentrations of the molecule also affected G0/G1 and G2/M populations.

Overall, these results indicate that 3-Cyclohexene-1-methanol exerts its antiproliferative effects by disrupting cell cycle progression in a concentration- dependent manner. As shown in Figure 5B, immunoblot analysis showed that this effect was associated with increased expression of cyclin-dependent kinase (CDK) inhibitor p21, activation of p53 at Serine 15 and decreased expression of CDK4 and the phosphorylated form of Rb in both cell lines regardless of p53 status. These findings suggest that 3-Cyclohexene-1-methanol modulates several regulatory proteins, leading to cell cycle arrest.

### 2.8. 3-Cyclohexene-1-Methanol Compound Induces Apoptosis of LS174 and HT29 Cells

**Figure 6:**
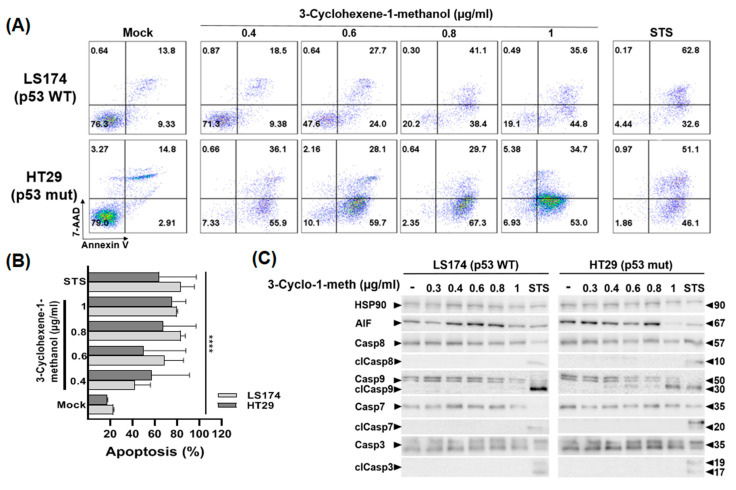
3-Cyclohexene-1-methanol induces apoptosis of LS174 and HT29 colon cancer cells regardless of p53 status. Exponentially growing LS174 and HT29 cells were treated with increasing concentrations of 3-Cyclohexene-1-methanol (0.4, 0.6, 0.8, and 1 µg/mL)) or with vehicle (DMSO) for 72 h. (**A**) Apoptosis was assessed by flow cytometry following Annexin V/7-AAD staining. Staurosporin (1 µM) was used as a positive control for apoptosis induction. Representative flow cytometry dot plots show the distribution of viable (Annexin V^−^/7-AAD^−^), early apoptotic (Annexin V^+^/7-AAD^−^), and late apoptotic (Annexin V^+^/7-AAD^+^) or necrotic (Annexin V^−^/7-AAD^+^) cell populations. (**B**) Quantitative analysis of apoptosis: the histogram illustrates the combined percentages of early and late apoptotic cells under each treatment condition. (**C**) Whole cell lysates were analyzed on SDS-PAGE gel and probed with the indicated antibodies for Western blot analysis. HSP90 was used as a control for equal loading Data are presented as mean ± standard error (SE) from three independent experiments (**** *p* < 0.0001 vs. control).

To investigate the mechanisms of cell death induced by 3-Cyclohexene-1-methanol, we assessed its ability to induce apoptosis in colon cancer cells using the Annexin V binding assay. Compared to mock-treated cells, no significant effect was observed after 24 h treatment (Figure S2). However, after 72 h of treatment, the compound triggered cell apoptosis with comparable effects in both colon cancer cells regardless of p53 status (Figure 6A,B). Interestingly, HT29 cells (p53 mut) were particularly sensitive to the 3-Cyclohexene-1-methanol treatment with more than 92% of cells undergoing apoptosis (early + late) even at the 0.4 µg/mL. At concentrations of 0.6 µg/mL, 0.8 µg/mL, and 1 µg/mL, the percentages of apoptotic cells were 87.8%, 97%, and 87.7%, respectively.

In LS174 cells (p53 wt), apoptosis levels reached 27.9%, 51.7%, 79.5%, and 80.4% at 0.4, 0.6, 0.8, and 1 µg/mL, respectively. Western blot analysis revealed that 3-Cyclohexene-1-methanol induced the cleavage of caspase-9 in HT29 cells starting at 0.4 µg/mL, indicating a caspase-dependent apoptosis pathway (Figure 6C). Notably, this effect was not associated with the activation of caspases-3, -7, or -8 nor with increased protein levels of the Apoptosis-inducing factor (AIF).

In LS174 cells, increased expression of AIF was observed at concentrations of 0.4, 0.6, and 0.8 µg/mL, indicating the induction of apoptosis via a caspase-independent pathway.

Overall, these results suggest that 3-Cyclohexene-1-methanol induces apoptosis in colon cancer cells via a caspase-independent pathway in LS174 cells (p53 wt) and a caspase-dependent mechanism in HT29 cells (p53 mut).

### 2.9. 3-Cyclohexene-1-Methanol Compound Modulates Survival-Signaling Pathways in LS174 and HT29 Colon Cancer Cells

**Figure 7:**
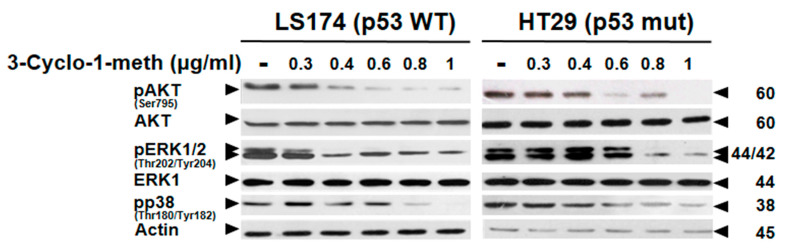
3-Cyclohexene-1-methanol modulates ERK1/2, p38 and AKT kinases in LS174 and HT29 cells. LS174 and HT29 cells were cultured under exponential growth conditions and treated with increasing concentrations of 3-Cyclohexene-1-methanol (0.3, 0.4, 0.6, 0.8, and 1 µg/mL) or vehicle for 72 h. Whole-cell lysates were prepared, subjected to SDS-PAGE, and analyzed by immunoblotting with the indicated specific antibodies. Actin served as a loading control to ensure equal protein loading.

To further decipher the mechanisms underlying the 3-Cyclohexene-1-methanol-inhibitory effect on the viability of HT29 and LS174 cells, we examined its impact on the phosphorylation status of the pro-survival kinases AKT, ERK1/2, and p38. As shown in Figure 7, immunoblot analysis revealed a progressive inhibition of AKT phosphorylation starting at 0.4 µg/mL in LS174 cells and at 0.6 µg/mL in HT29 cells. Moreover, 3-Cyclohexene-1-methanol induced the dephosphorylation of ERK1/2 kinases beginning at 0.4 µg/mL in LS174 cells and at 0.8 µg/mL in HT29 cells. Similarly, a reduction in p38 was observed starting at 0.4 µg/mL in LS174 cells and at 0.8 µg/mL in HT29 cells. These findings suggest that 3-Cyclohexene-1-methanol disrupts AKT, ERK1/2, and p38 signaling -key components of survival and proliferation pathways-thereby contributing to reduced cell viability, cell cycle perturbation and apoptosis in both cell lines.

## 3. Discussion

The fluorinated analog of uracil, 5-Fluorouracil (5-FU), remains the central therapeutic option in the management of colorectal cancer [28]. This drug is widely used both in the adjuvant setting to treat patients with early-stage CRC and in combination with chemotherapy regimens to manage metastatic CRC. Unfortunately, poor treatment outcomes have been observed, primarily due to the high rate of relapse caused by intrinsic or acquired resistance in patients’ tumors [29,30]. Therefore, there is still a need to develop alternative strategies that can bypass these limitations and improve the clinical outcomes of drug treatments. In this context, the potential of natural products in combating cancer has gained increasing attention. Eucalyptus essential oil (EEO) holds significant industrial importance [31] and its effectiveness against various diseases is well documented [32]. Indeed, EEO possesses a wide variety of interesting pharmacological bioactivities [31].

In this study, we investigated the anti-proliferative properties of a Tunisian EEO and its major compounds against colorectal cancer cells. We found that EEO gradually reduced the proliferation of LS174 colon cancer cells after 24 h of treatment. Based on cell viability and lactate dehydrogenase (LDH) assays, we selected an effective dose that was non-toxic to cells and did not affect the viability of non-tumorigenic cells for mechanistic investigations.

Due to the critical role of the MAPK and AKT signaling pathways in regulating cellular growth and survival in colorectal cancer [21,33,34], targeting their components is considered a valuable approach in the anticancer therapy. For this reason, we investigated whether EEO-induced inhibition of LS174 cell viability could affect ERK1/2, p38, JNKs and AKT kinases. Notably, we found that EEO-induced cell death in LS174 cells was associated with the inhibition of ERK1/2 and AKT, along with the activation of p38 and JNK kinases. Our data are in agreement with previous studies reporting an inhibition of ERK activity in colorectal cancer associated with reduced tumor growth [35]. Moreover, the inhibition of the AKT/ERK signaling pathway has also been reported to reduce the proliferation, migration, invasion, and angiogenesis in colon cancer cells [36]. Our data further support previous reports showing that modulation of the MAPK activity, through ERK inhibition and JNK activation, contributes to the induction of apoptotic pathways in intestinal cells [37]. Indeed, activation of JNK signaling has been shown to reduce the proliferation and promote cell death in colon cancer cell lines [38,39,40]. In agreement with our result, it has also been reported that many chemotherapeutic agents induce apoptosis in colon cancer cells by activating the p38 kinase [41,42,43,44].

It is well known that defects in the apoptotic pathway are a hallmark of cancer. For this reason, apoptosis remains a promising target for anticancer therapy [45]. Apoptosis can be triggered through two main pathways: the intrinsic (mitochondrial) and the extrinsic (death receptor) pathways [46,47]. The intrinsic pathway is triggered by intracellular stress signals, such as DNA damage, oxidative stress, or deprivation of growth factors. In this process, mitochondria play a central role in regulating the release of apoptogenic factors, such as cytochrome c. This release activates caspases, which ultimately lead to programmed cell death [48]. This pathway is tightly regulated by the balance between pro-apoptotic and anti-apoptotic members of the Bcl-2 protein family [45].

Our results revealed that EEO induced apoptosis in LS174 cells in a caspase-dependent mechanism. Caspase-8 is well known as an initiator of apoptosis while caspase-3 is the main executioner, and their activation is a central event in cell death. Interestingly, EEO induced the cleavage of caspase-3 and decreased the protein level of caspase-8. This modulation was associated with an increase in the level of cytochrome c, a key mediator in the mitochondrial pathway, [49]. Additionally, we found that EEO triggered two pro-apoptotic regulators by dephosphorylating BAD and upregulating TRADD expression. This is consistent with previous studies showing that TRADD plays a role in cell death through FADD and caspase 8 recruitment [50,51,52,53].

Bad is a member of the Bcl-2 family and typically binds to the Bcl-2/Bcl-X complex to induce apoptosis. Upon phosphorylation, Bad dissociates from this complex, leading to the release of Bcl-2/Bcl-XL and the inhibition of apoptosis [54]. We found that EEO reduced the phosphorylation of Bad. This effect was associated with increased BAX mRNA expression and elevated levels of the p53 upregulated modulator of apoptosis (PUMA) protein which promote apoptosis partly by displacing Bax from Bcl-XL, thereby facilitating Bax multimerization and mitochondrial translocation [55]. These events trigger cytochrome c release and subsequently activate programmed cell death. Our results indicate that EEO initiates cell apoptosis via the caspase-dependent mitochondrial pathway. These findings are in concordance with previous studies demonstrating that EEOs and their components induce apoptosis in RKO and SW48 colon cancer cells [19,56].

Aberrant cell cycle progression plays a crucial role in tumor cell proliferation [57]. Therefore, targeting this process represents a promising anticancer strategy [58]. Interestingly, we found that EEO arrested LS174 colon cancer cells in the G2/M phase. Elucidating the molecular mechanisms underlying this effect is key to understanding how this natural compound exerts its activity.

A cyclin-dependent kinase (CDK) inhibitor, the p27 protein, induces cell cycle arrest by binding to cyclin–CDK complexes [59,60]. Specifically, p27 inhibits CDK2 and CDK4/6 thereby preventing the phosphorylation of the Retinoblastoma [61,62]. In its hypophosphorylated state, pRb binds to E2F transcription factor and blocks the expression of genes required for entry to the S phase, thus maintaining the cell in G1 arrest. Conversely, when p27 levels decrease, CDKs become active, leading to the hyperphosphorylation of Rb, release of E2F and cell cycle progression [63,64]. Additionally, Cyclin D1, in conjunction with CDK4/6, plays a crucial role in initiating the phosphorylation of Rb [65]. In this study, we found that EEO increased the expression of the p27 protein while decreasing the phosphorylated form of Rb and inhibiting Cyclin D1, ultimately leading to cell cycle arrest.

Furthermore, treatment of LS174 cells with EEO resulted in an overexpression of the p53 tumor suppressor, which can subsequently lead to cell cycle arrest [66,67]. The primary mechanism by which p53 induces cell cycle arrest is through the transcriptional downregulation of various cell cycle related genes [68]. Among these are CDC2 and CDK2, which are involved in the G1/S and G2/M transitions [68]. Interestingly EEO induced cell cycle arrest by decreasing the expression of CDK2 protein and dephosphorylating CDC2. Together, these results indicate that EEO plays a pivotal role in modulating cell cycle progression by regulating several effectors including Cyclin D1, p53, CDK2, CDC2, Rb, and p27 in colorectal cancer cells.

In line with the literature, the analysis of the chemical composition of EEO revealed the presence of Eucalyptol (also called 1,8-cineole), as the predominant compound, accounting for 63.28% of the total composition. Numerous studies have reported a high percentage of Eucalyptol in the essential oils of various Eucalyptus species [69] in *E. saligna* (61.3%) (Democratic Republic of the Congo) [70], *E. globulus* (90.0%) (Australia) [71], *E. camaldulensis* (74.7%) (Iran) [72], *E. citriodora* (54.1%) (Tunisia) [73], *E. cinerea* (88.5%) (Argentina) [74].

We also identified three other major compounds in EEO: 3-Cyclohexene-1-methanol (10.18%), α-Pinene (9.19%), and α-terpineol (4.95%). Several reports have indicated comparable levels of α-Pinene and α-terpineol in essential oils from certain Eucalyptus species [75,76]. In contrast, this is the first report identifying 3-Cyclohexene-1-methanol as one of the major constituents of Tunisian EEO. This finding may be explained by the high variability in the chemical composition of essential oil, which can be influenced by exogenous factors such as precipitation, soil type, temperature, altitude, and light exposure. This variability could also be linked to endogenous factors including the plant’s anatomical, physiological, and genetic characteristics, which regulate essential oil biosynthesis. Additionally, environmental conditions can affect the DNA of aromatic plants, leading to genotype variations [76,77].

Studying individual compounds within a whole extract as potential anti-tumor agents is crucial for identifying the specific bioactive molecules responsible for the beneficial effects. A total extract is a complex mixture containing active and inactive components [78], which may have antagonistic effects [79,80]. Isolating and characterizing the active compounds allows a better understanding of their precise molecular mechanisms [81]. In addition, working with isolated compounds ensures reproducibility and standardization, as the composition of total extracts can vary significantly depending on extraction conditions and biological sources [82,83]. Identifying active molecules also enables chemical modifications aimed to improve their pharmacological properties. However, the pharmaceutical development and successful therapeutic application of bioactive compounds from natural products are often challenged by their pharmacokinetics, particularly poor bioavailability. In this context, nanoformulation strategies have attracted considerable attention as a means to enhance their solubility, efficacy, bioavailability, deliver the compound directly to the target tumor microenvironment site, increase local drug concentrations and minimize systemic toxicity [10,84,85].

In this study, we investigated the role of the p53 protein in the pharmacological activity of the four major compounds of EEO using two colorectal cancer cell lines: LS174 (wild-type p53) and HT29 (mutated p53). Approximately 60% of colorectal cancers harbor mutations in the tumor suppressor gene encoding p53 [86,87]. These mutations lead to the functional inactivation of the p53 protein and are consequently associated with therapeutic failure due to the resistance of p53 mutant or deficient tumors to therapies [88]. Our results demonstrated that Eucalyptol, 3-Cyclohexene-1-methanol, α-Pinene, and α-terpineol effectively inhibited the proliferation of colorectal tumor cells, irrespective of their p53 status. These findings align with various studies reporting anti-tumor properties of some compounds identified in the chemical composition of EEO. Notably, 1,8-cineole (Eucalyptol) exhibited an antiproliferative effect on two colorectal HCT116 and RKO cancer cells via the modulation of survivin, and the activation of Akt and p38 pathways. The anti-tumor activity of Eucalyptol has been associated with the induction of cell apoptosis through Poly(ADP-ribose) polymerase (PARP) cleavage and the activation of caspase-3 [56] as well as inhibition of colorectal tumor cell viability through suppression of NF-κBp65/JAK and Bcl-2/Caspase signaling pathways [89].

The α-Pinene component inhibited the proliferation of colon cancer cells, including HT29, Caco-2, and CT26 [90]. It also exhibited antiproliferative activity in hepatocellular carcinoma (HepG2) cells [91] and hepatoma carcinoma BEL-7402 cells, inducing G2/M cell cycle arrest by upregulating Chk1 and Chk2 while downregulating Cyclin B, CDC25, and CDK1 [92]. Several studies have reported that α-Terpineol acts as an anticancer agent and induces tumor cell death in small cell lung carcinoma by inhibiting NF-κB activity [93]. Moreover, α-Terpineol-loaded PLGA nanoparticles coated with folic acid and chitosan demonstrated a pro-apoptotic effect in HT-29 cells by increasing the percentage of cells in the SubG1 phase and modulating Bax and Bcl-2 [11].

To the best of our knowledge, the inhibitory effect of 3-Cyclohexene-1-methanol on the viability of tumor cells has not been previously investigated. Interestingly, we demontrated for the first time that this compound exerts antiproliferative activity against both HT29 and LS174 colon cancer cells irrespective of their p53 status. This effect was associated with the induction of cell cycle arrest.

Tumor suppression and cell cycle regulation are closely linked to the p53-p21-Rb axis [67]. P21 is a target of p53 and exerts its tumor suppressive function by activating p53 through post-translational modifications such as phosphorylation and inhibiting the activity of cyclin–CDK complexes, thereby halting cell proliferation in response to cellular stress or damage [67]. In this study, 3-Cyclohexene-1-methanol increased the expression of p21, a key regulator well known for its pivotal role in controlling cell cycle progression at the G1/S and G2/M checkpoints [67]. The upregulation of this p21 together with the activation of p53 led to a concomitant downregulation of several cell cycle regulators such as pRb and CDK4 resulting in cell cycle arrest. In line with our data, it has been reported that the essential oil of *Tarchonanthus camphoratus*, whose major constituent is 3-Cyclohexene-1-methanol, inhibited the viability of MCF-7 cells by inducing cell cycle arrest [94]. In agreement with our data, it has been shown that the p53 status of target cells can influence how therapeutic agent affects cell cycle progression. In fact, treatment of two cell lines (one expressing wild-type p53 and the other harboring mutant p53) with the same anticancer compound may lead to arrest at different phases of the cell cycle [95]. In cells with functional p53, treatment typically induces G1 phase arrest, through the upregulation of p21, which inhibits cyclin-dependent kinases and prevents progression to the S phase [96]. In contrast, in cells with mutant or inactive p53, the G1 checkpoint is compromised, and arrest commonly occurs at the G2/M transition, relying on p53-independent pathways involving Chk1/Chk2 [97,98]. We found that 3-Cyclohexene-1-methanol induces apoptosis of colorectal HT29 and LS174 cancer cells through distinct mechanisms that rely on the compound concentration. In HT29 cells, it activated caspase-9 starting at a concentration of 0.4 µg/mL without concomitant activation of caspases-3, -7, or -8. This indicates a caspase-9–dependent apoptotic pathway, likely involving intrinsic mitochondrial signaling rather than the classical executioner caspases [99]. In agreement, previous reports have shown that caspase-9 can induce lysosomal cell death in a manner independent of caspases-3 and -7 [100]. In contrast, treatment with 3-Cyclohexene-1-methanol (at 0.4 to 0.8 µg/mL) induced apoptosis in LS174 cells that appears to be mediated by the AIF protein suggesting a caspase-independent mechanism [101]. Such response is often observed in cells under severe oxidative or mitochondrial stress [101,102]. These findings suggest that 3-Cyclohexene-1-methanol, differentially activates apoptotic pathways depending on the cell line [103]. This compound exhibited its activity by modulating the activation of ERK, AKT and p38 kinases in both colorectal tumor cells.

It is well known that the two major signaling pathways (ERK and p38 MAPK and the PI3K/AKT) are constitutively activated in colorectal cancers due to genetic alterations [104] and are regulated by receptor tyrosine kinases (RTKs) and heterotrimeric G-protein-coupled receptors (GPCR). Modulation of these pathways by therapeutic molecules can result in different cellular outcomes depending on the p53 status [105,106].

In agreement with the literature, we propose that 3-Cyclohexene-1-methanol compound regulates cell proliferation and survival by modulating these kinase pathways potentially through RTK, GPCR, or other upstream effectors.

## 4. Materials and Methods

### 4.1. Plant Material and Pure Compounds

The commercial Eucalyptus essential oil used in this study was purchased from Lamia Cherni farm (Dahmani, Tunisia). It was extracted from the leaves of *Eucalyptus globulus* located in the northwestern region of Tunisia through a meticulous hydro-distillation process according to the standard procedure described in the European Pharmacopoeia.

The four major pure compounds identified in the Eucalyptus essential oil were purchased from Sigma-Aldrich (St. Louis, MS, USA). Their characteristics are listed as follows: α-Pinene (Purity: 98%, Density: 0.858 g/mL at 25 °C, Formula: C_10_H_16_, Molecular Weight: 136.23 g/mol, Ref: #147524), Eucalyptol (1,8-Cineole) (Purity: 99%, Density: 0.921 g/mL at 25 °C, Formula: C_10_H_18_O, Molecular Weight: 154.25 g/mol, Ref: #C80601), α-Terpineol (Purity: 90%, Density: 0.93 g/mL at 25 °C, Formula: C_10_H_18_O, Molecular Weight: 154.25 g/mol, Ref: #432628), and 3-cyclohexene-1-methanol (Purity: 98%, Density: 0.961 g/mL at 25 °C, Formula: C_6_H_9_CH_2_OH, Molecular Weight: 112.17 g/mol, Ref: #162167).

### 4.2. Cell Culture

Human colon adenocarcinoma LS174 and HT29 cell lines as well as Human Embryonic Kidney 293 cells (HEK293) were obtained from ATCC (American Type Culture Collection, Manassas, VA, USA). Cells were routinely tested for mycoplasma contamination, and cultured in a DMEM medium (Dulbecco’s Modified Eagle’s Meduim, GIBCO, Invitrogen, Carlsbad, CA, USA) supplemented with 10% fetal bovine serum (FBS; GIBCO). Cultures were incubated in a humidified atmosphere with 5% CO_2_ and 95% air at 37 °C. All experiments were performed 24 h after seeding, after which cells were treated with various concentrations of EEO or pure compounds.

### 4.3. Cell Viability

Cell viability was investigated by measuring mitochondrial function using the 3-(4,5-dimethylthiazol-2-yl)-2,5-diphenyl-tetrazolium bromide (MTT, Sigma-Aldrich, St. Louis, MS, USA). Briefly, 1000 cells/well were seeded in 96-well plates for 24 h. Cells were then treated with increasing concentrations of EEO or pure compounds. After 24 h or 72 h the medium was replaced with fresh one (100 µL/well) containing MTT (1 mg/mL), and the cells were incubated for another 3 h at 37 °C. The crystals formed after the reduction of MTT by mitochondrial dehydrogenases were dissolved by adding 100 µL of dimethyl sulfoxide (DMSO, Sigma-Aldrich, St. Louis, MS, USA). Finally, using a microplate reader (Labsystems MULTISCAN, Thermo Fisher Scientific, Waltham, MA, USA), the optical density (OD) was measured at 540 nm. The relative number of viable cells was compared to mock-treated cells (control), and the cell viability was calculated using the formula: Cell viability (%) = OD test/OD control × 100.

### 4.4. Cell Cytotoxicity

Cell cytotoxicity was measured by lactate dehydrogenase (LDH) release activity using the LDH Cytotoxicity assay kit (Roche Boehringer Mannheim, Rueil-Malmaison, Hauts-de-Seine, France) following the manufacturer’s instructions. Briefly, cells were seeded at a density of 5 × 10^5^ cells per well. After 24 or 72 h of treatments, 100 µL of each treated condition’s supernatant was collected for the LDH assay. Absorbance was measured at 492 nm. The intensity of the generated color correlated directly with the number of lysed cells. The percentage of LDH release was calculated by comparing the release from treated cells to that of the positive control (1% Triton X-100 treatment, 100% toxicity) and the negative control (mock-treated cells with spontaneous LDH release) as follows:

Cytotoxicity (%) = (experimental value-mock negative control)/(positive control-negative control) × 100.

### 4.5. Cell Cycle Analysis

The cell cycle analysis was performed by quantifying DNA content using propidium iodide (PI) staining. Briefly, tumor cells were plated in six-well plates and then treated with EEO (0.2 µg/mL) or increasing concentrations of the selected compound for 24 h and/or 72 h. After treatments, cells were centrifuged at 1000 rpm and washed twice with ice-cold PBS containing 2% bovine albumin serum (BSA). To permeabilize the cell membrane, 500 µL of Hypertonic-Triton Buffer (containing 20 mM HEPES, pH 7.2; 0.16 M NaCl; 1 mM EGTA; and 0.05% Triton X-100) was added, followed by rapid incubation on ice for 1 min. Then, cells were resuspended in propidium iodide (PI)/RNase staining solution (Cell Signaling Technology; Danvers, MA, USA) and incubated for 30 min in the dark. The cell cycle progression was assessed using a Becton Dickinson FACScanto II flow cytometer (BD Biosciences, San Jose, CA, USA) and further analyzed with BD FACS Diva 6 software (BD Biosciences, San Jose, CA, USA) as well as Flow Jo software v10 (BD Biosciences, USA). The PI fluorescence signal at the FL2-A peak versus counts was utilized to determine the distribution of cells in the cell cycle.

### 4.6. Assessment of Cell Apoptosis

For apoptosis analysis, Annexin V-PE/7-amino-actinomycin D (7-AAD) staining using an Annexin V-PE apoptosis detection kit (BD Biosciences, San Jose, CA, USA) was performed by flow cytometry according to the manufacturer’s guidelines. Colon cancer cells were treated with EEO (0.2 µg/mL) or increasing concentrations of the selected compound. After 24 h and/or 72 h at 37 °C, cells were stained with Annexin V-PE/7-AAD and followed by incubation for 15 min in the dark. The fluorescence was measured by a Becton Dickinson FACScanto II flow cytometer (BD Biosciences, San Jose, CA, USA), and the results were analyzed with BD FACSDiva 6 software (BD Biosciences, San Jose, CA, USA) as well as Flow Jo software v10 (BD Biosciences, San Jose, CA, USA). According to the method, Annexin V-PE (-)/7-AAD (-) indicates viable cells, Annexin V-PE (+)/7-AAD (-) indicates cells of apoptosis in the early stage, and cells in late-stage of apoptosis (Annexin V-PE (+)/7-AAD (+)) or necrosis were labeled Annexin V-PE (-)/7-AAD (+). The percentage of apoptotic cells (%) was calculated as follows: Early apoptotic cells (%) + late apoptotic cells (%).

### 4.7. Western Blotting Analysis

Sodium Dodecyl Sulfate-Polyacrylamide Gels (SDS-PAGE) and immunoblotting analyses were performed using standard protocols [7]. After treatments with vehicle (mock), EEO (0.2 µg/mL) for 24 h or increasing concentrations of the selected compound for 72 h, cells were washed twice with PBS and lysed in Laemmeli buffer at room temperature. The concentration of total proteins was measured using the Bicinchoninic acid (BCA) protein assay. Equal amounts of cell lysates (30 µg) were resolved on SDS-PAGE and transferred onto polyvinylidene fluoride membranes (PVDF). Then, membranes were blocked for 1 h in the blocking buffer containing 5% non-fat dry milk at room temperature. Thereafter, membranes were incubated overnight with indicated primary antibodies (anti-p53, anti-pp53 (Ser15), anti-p27, anti-p21, anti-Cyclin D1, anti-CDK2, anti-CDK4, anti-phospho CDC2 (Tyr15), anti-phospho Rb, anti-TRADD, anti-Caspase 8, anti-Caspase 7, anti-Caspase 9, anti-AIF, anti-Caspase 3, anti-Bad, anti-phospho Bad (Ser112), anti-Cytochrome C, anti-Bik, anti-Bax, anti-Puma, anti-AKT, anti-phospho AKT (Ser473), anti-phospho ERK1/2 (Thr202/Tyr204), anti-ERK1/2, anti-phospho p38 (Thr180/Tyr182), anti-p38, anti-phospho SAPK/JNK (Thr183/Tyr185), anti-HSP90, anti-β-actin) from cell Signaling Technology (Danvers, MA, USA) at a dilution of 1:1000. β-actin and HSP90 were used as the loading control. Immunoreactivity was detected by using horseradish peroxidase-conjugated anti-rabbit or anti-mouse IgG (Promega, Madison, WI, USA) and visualized in an X-ray film or using a ChemiDoc MP Imaging System (Bio-Rad, Hercules, CA, USA) after incubation with enhanced Chemiluminescent (ECL, Pierce, Rockford, IL, USA).

### 4.8. Real-Time Quantitative RT-PCR

The expression of several genes involved in cell cycle progression and apoptosis was assessed by a real-time quantitative RT-PCR. Mock or EEO-treated-LS174 cells (0.2 µg/mL) were lysed after 24 h using TRIzol (Invitrogen, Carlsbad, CA, USA). Total RNA was extracted using RNeasy Mini Kit (QIAGEN, Hilden, Germany) according to the manufacturer’s protocol. The cDNA was prepared from reverse transcribed RNA (2 µg) using oligo (dT) and Superscript III reverse transcriptase (Invitrogen, Carlsbad, CA, USA). Real-time PCR was performed in an ABI PRISM 5700 Sequence Detector System (Applied Biosystems, Courtaboeuf, France) using the SYBR Green Master mix (Applied Biosystems, Courtaboeuf, France) detection protocol. The relative expression of target transcripts was calculated using the 2^[−ΔΔC (T)]^ method after normalization with four housekeeping genes (GAPDH, β-actin, HPRT and ubiquitin) as previously described [103].

### 4.9. Chemical Characterization of Eucalyptus Essential Oil (EEO) and Compounds Identification

The chemical analysis of commercial *Eucalyptus globulus* essential oil (EEO) was performed by gas chromatography (GC; Agilent 6890N, Agilent Technologies, Paris, France) combined to mass spectroscopy (MS; Agilent 5973N, Agilent Technologies) as previously described by Mathlouthi et al. [107]. The capillary column used was the HP5-MS 5% phenyl methyl siloxan (Length: 30 m; internal diameter: 0.25 mm; film thickness: 0.25 µm) and an automatic passer (Agilent 7683B; Agilent Technologies). Helium was the carrier gas at a flow rate of 1 mL/min. The column temperature was initially adjusted at 5 °C for 1 min then increased progressively at a rate of 2 °C/min to reach 300 °C within 130 min. The samples were diluted in ethanol (1/10) then 1 μL was injected into GC-MS. The parameters used were listed as follows: Injection temperature: 260 °C; interface heating: 300 °C; ion source heating: 200 °C; Ionization Energy voltage: 70 eV; scan range: 41–450 amu; and scan time 0.50 s. The Oven temperature programmes were as follows: (i) 55–120 °C (3 °C/min), 120–200 °C (4 °C/min), 200–220 °C (6 °C/min) and 220 °C for 5 min, and (ii) 60–240 °C at 3 °C/min, carrier gas He, 54.8 kPa. The components were identified by comparing their relative retention times and mass spectra with the standard data (NIST05, Mass Spectra Library, National Institute of Standards and Technology, Gaithersburg, MD, USA).

### 4.10. Statistical Analysis

Statistical analysis was performed using GraphPad Prism v8 (GraphPad Software, San Diego, CA, USA). Data from each experiment were expressed as the mean ± SE of duplicates from three independent experiments. Data were analyzed using one-way ANOVA or Student’s *t*-test. Differences were considered to be statistically significant at *p* < 0.05 (* *p* < 0.05, ** *p* < 0.01, *** *p* < 0.001, *** *p* < 0.0001).

## 5. Conclusions

**Figure 8:**
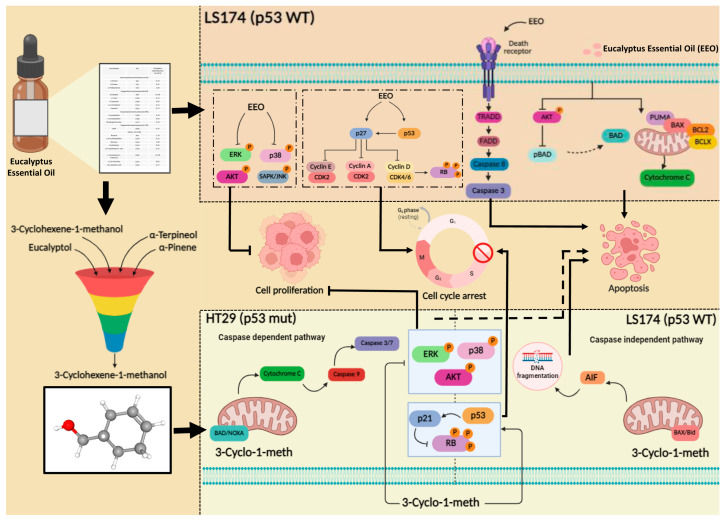
Schematic model summarizing the mechanisms underlying the anti-tumor effects of Eucalyptus essential oil and its component, 3-Cyclohexene-1-methanol, against colorectal cancer cells. (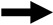 Activation 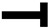 Inhibition).

Our results showed that EEO exerted an antitumor effect on colon cancer cells in vitro by reducing cell viability, inducing apoptosis, cell cycle arrest and modulating key survival signaling pathways. Moreover, the major bioactive compounds of EEO significantly reduced the proliferation of LS174 and HT29 cells regardless of their p53 status. Among them, 3-Cyclohexene-1-methanol exhibited the most pronounced antiproliferative activity by affecting cell cycle progression and inducing apoptotic cell death through regulating several cellular effectors involved in these processes. Overall, our findings suggest that Eucalyptus essential oil and, in particular, its major component, 3-Cyclohexene-1-methanol, may serve as multi-targeting-agents with beneficial properties against tumor cells (Figure 8). These are promising candidates for colorectal cancer management that could be used alone or in combination with therapeutic drugs.

However, despite the interesting properties of bioactive compounds, the biological variability in potency poses significant challenges in the development of effective therapeutics primarily due to limited drug solubility and bioavailability.

Nowadays, nanotechnology and biotechnology represent a cutting-edge frontier in pharmaceutical sciences that could pave the way for harnessing the full potential of natural compounds, making them integral to the future of cancer research and personalized medicine.

To consolidate these results, further pharmacokinetic and toxicological studies are warranted to validate the stability and safety of 3-Cyclohexene-1-methanol before expanding its potential for a successful therapeutic application.

## Figures and Tables

**Table 1:** Chemical composition of Eucalyptus Essential Oil.

Constituents	RI	% Value
Monoterpene hydrocarbons (10.5%)		
α-Pinene	887	9.19
β-Pinene	932	0.25
α-Phellandrene	970	1.06
Oxygenated monoterpenes (68.6%)		
Eucalyptol	999	63.28
z-Citral	1439	0.12
α-Terpineol	1490	4.95
2,6-Octadienal	1520	0.12
Geraniol	1643	0.13
Sesquiterpene hydrocarbons (0.89%)		
Caryophyllene	1380	0.44
α-Caryophyllene	1448	0.04
Bicyclogermacrene	1511	0.41
Oxygenated sesquiterpenes (0.15%)		
Ledol	1849	0.15
Others (14.35%)		
Butanal	842	1.29
1,4-Cyclohexadiene	1039	0.32
Benzene	1063	1.08
Cyclohexene	1074	0.45
1H-Cyclohexene-1-one	1476	0.21
3-Cyclohexene-1-methanol	1487	10.18
1,3,6-Octariene	1035	0.65
Cis-p-Mentha-1(7)	1585	0.17

Compounds were identified by GC-MS. Their retention indices (RI) were determined relative to homologous series of n-alkanes. The compound distribution in different chemical classes is presented.

**Table 2:** IC_50_ values of the four major compounds identified in EEO (in µg/mL).

Cell Lines	LS174 (p53 WT)	HT29 (p53 Mut)
Compounds	IC_50_ (µg/mL)	
	24 h	72 h
α-Pinene	>2.72	1.05
Eucalyptol	3.09	1.13
α-Terpineol	0.85	0.93
3-cyclohexene-1-methanol	0.6	0.30

HT29 and LS174 colorectal cancer cells were treated with different concentrations of the major components identified in EEO for 24 h or 72 h. Cell viability was assessed using the MTT assay. IC_50_ was calculated using GraphPad Prism v8 (GraphPad Software, San Diego, CA, USA).

## Data Availability

All data supporting the findings of this study have been included in the manuscript.

## Supplementary Materials

The following supporting information can be downloaded at: https://www.mdpi.com/article/10.3390/ijms26188876/s1.
