# Supplementary material for: Deciphering the Mechanisms Underlying the Antitumor Effects of Eucalyptus Essential Oil and Its Component 3-Cyclohexene-1-Methanol Against Human Colon Cancer Cells
Source: Int J Mol Sci. 2025 Sep 12;26(18):8876. doi: 10.3390/ijms26188876 (PMC12469830; doi:10.3390/ijms26188876)
Supplement: Supplementary file 1 [file ijms-26-08876-s001.zip › ijms-3806022-supplementary.pdf]

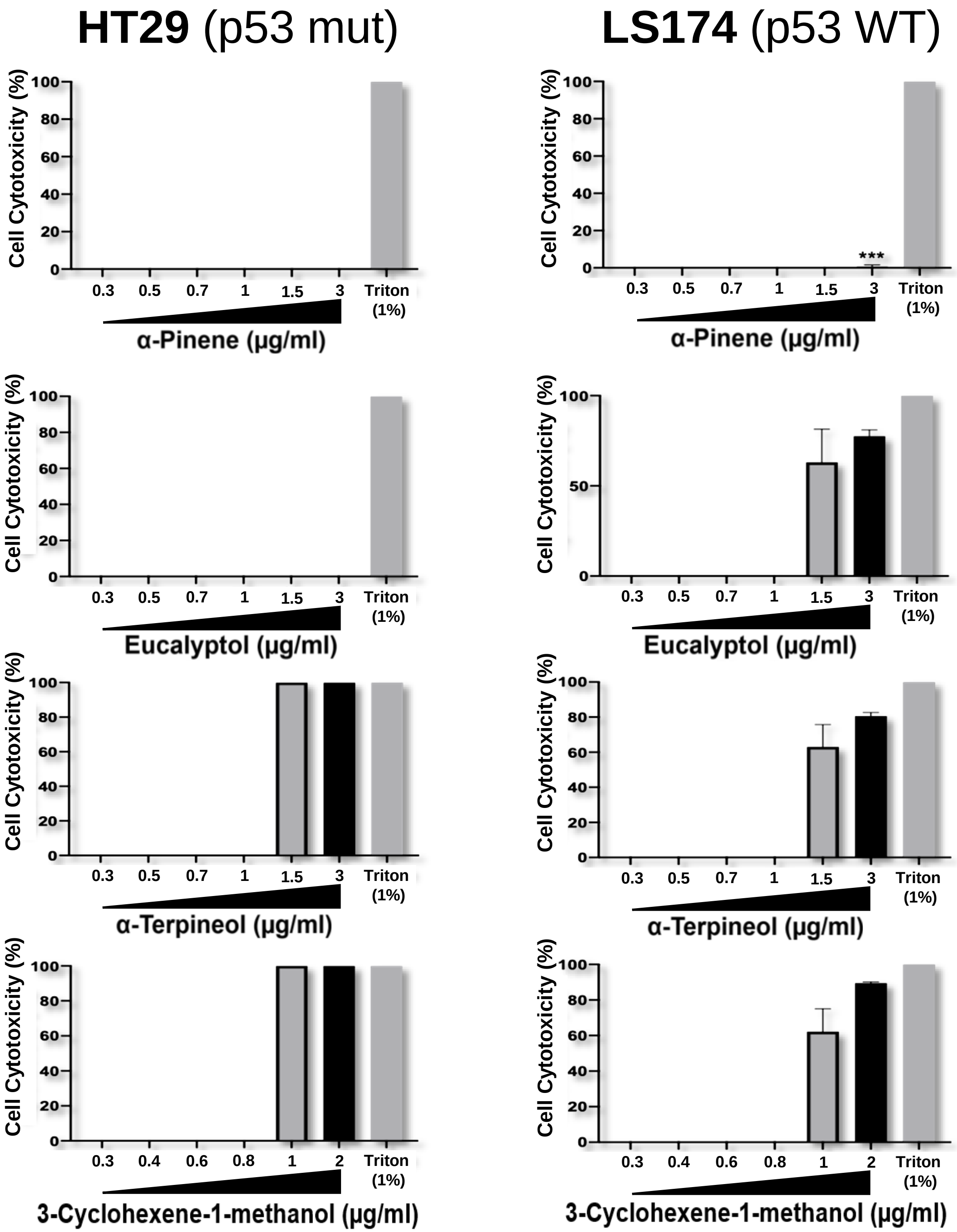

Figure S1. The four major compounds of Eucalyptus essential oil (EEO) inhibit the proliferation of LS174 and HT29 colon cancer cells independently of their p53 status. LS174 and HT29 cells were treated for 24 h with increasing concentrations of α-pinene, eucalyptol, α-terpineol, and 3-Cyclohexene-1-methanol or vehicle (DMSO). Cytotoxicity was evaluated by lactate dehydrogenase (LDH) release assay. LDH activity was expressed as a percentage of the release induced by 1% Triton X-100 (100% cyototoxicity). Data represent the mean ± standard error (SE) of three independent experiments. Statistical significance was determined using Student’s t-test (*** $p < 0.0001$ vs. control).

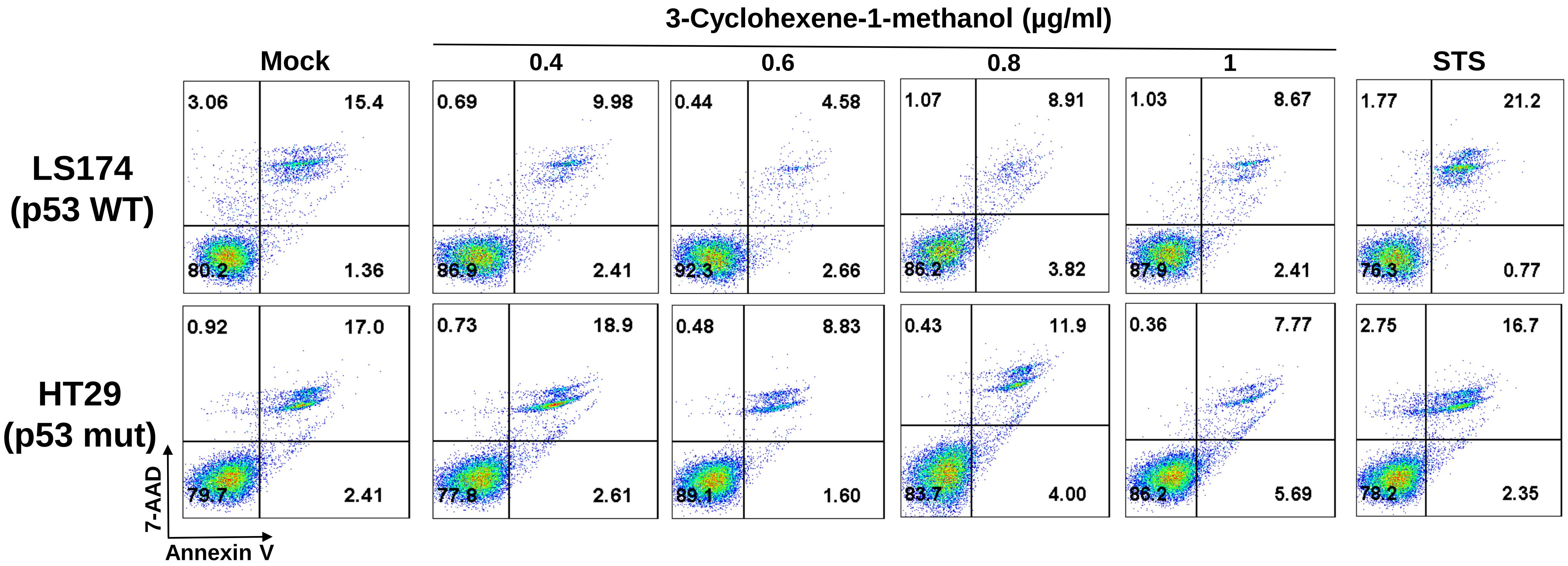

Figure S2. 3-Cyclohexene-1-methanol induces apoptosis of LS174 and HT29 colon cancer cells regardless of p53 status. Exponentially growing LS174 and HT29 cells were treated with increasing concentrations of 3-Cyclohexene-1-methanol (0.4, 0.6, 0.8, and 1 µg/mL)) or with vehicle (DMSO) for 24 h. Apoptosis was assessed by flow cytometry following Annexin V/7-AAD staining. Staurosporin (1 µM) was used as a positive control for apoptosis induction. Representative flow cytometry dot plots show the distribution of viable (Annexin V-/7-AAD-), early apoptotic (Annexin V+/7-AAD-), and late apoptotic (Annexin V+/7-AAD+) or necrotic (Annexin V-/7-AAD+) cell populations.
